# The Medical Review (Medical and Surgical Review of Reviews)

**Published:** 1900-03

**Authors:** 


**The Medical Review (Medical and Surgical Review of Reviews).** Edited by NATHAN E. BOYD, M.D. London: Connaught Mansions.

The second volume, January—December, 1899, contains 768 double pages, forming an indexed and illustrated monthly summary of all that is important to the practitioner in the medical periodicals of the world. The following quotation from the title page indicates the aims and uses of this handsome volume:

"By the suppression of all unessential matter, a paper written with any definite object—and such alone is valuable—can generally be compressed into a comparatively brief report, and yet remain a clear and readable account of the subject, so that nothing of importance is lost, and often, in lucidity, much is gained. Merely to mention a few of the leading features of an article serves no useful purpose."

